# Online Support and Intervention for Child Anxiety (OSI): Development and Usability Testing

**DOI:** 10.2196/29846

**Published:** 2022-04-13

**Authors:** Claire Hill, Tessa Reardon, Lucy Taylor, Cathy Creswell

**Affiliations:** 1 School of Psychology & Clinical Language Sciences University of Reading Reading United Kingdom; 2 Department of Experimental Psychology University of Oxford Oxford United Kingdom; 3 Department of Psychiatry University of Oxford Oxford United Kingdom

**Keywords:** user-centered design, co-design, usability testing, internet-based treatment, app, CBT, anxiety, children, mobile phone

## Abstract

**Background:**

Internet-based treatments for child anxiety may help to increase access to evidence-based therapies; however, user engagement, uptake, and adherence within routine clinical practice remain as challenges. Involving the intended end users in the development process through user-centered design and usability testing is crucial for maximizing user engagement and adoption of internet-based treatments, but so far this has been lacking for internet-based treatments for child anxiety.

**Objective:**

The aim of this study is to develop an internet-based treatment for child anxiety through a process of user-centered design (phase 1) and usability testing (phase 2), based on an existing evidence-based, face-to-face, therapist-supported, parent-led cognitive behavioral therapy intervention. It is intended that the internet-based version of this treatment would consist of a parent website, case management system for clinicians, and mobile game app for children.

**Methods:**

Parents, children, and clinicians who were familiar with the face-to-face version of the treatment were recruited from 2 National Health Service clinics. In phase 1, participants participated in 3 workshops to gain feedback on the overall concept, explore their wants and needs for the websites and game, generate ideas on how the treatment may look, and gain feedback on initial mock-ups of the websites and game. In phase 2, participants attended 3 individual usability testing sessions where they were presented with working prototypes of the website or game and asked to perform a series of tasks on the website (parents and clinicians) or play the game (children). The frequency and details on usability errors were recorded. Participants were asked for their feedback on the website and game using a standardized usability questionnaire and semistructured interviews. The websites and game were iterated after each round of usability testing in response to this feedback.

**Results:**

In phase 1, participants approved the general concept and rated the initial mock-ups of the website and game positively. In phase 2, working prototypes were rated positively and usability errors declined across the iterations and were mainly cosmetic or minor issues relating to esthetic preference, with few issues regarding ability to navigate the website or technical issues affecting functionality. Feedback from the semistructured interviews further supported the positive response of participants to the website and game, and helped identify areas for improvement during the iteration process. The final iteration of the website and game are presented.

**Conclusions:**

Taking an iterative approach to development through user-centered design and usability testing has resulted in an internet-based treatment for child anxiety (Online Support and Intervention for child anxiety) that appears to meet the needs and expectations of the intended users (parents, children, and clinicians) and is easy and enjoyable to use.

## Introduction

### Background

There is growing interest in providing psychological treatments via the internet to increase access to evidence-based therapies. This is particularly salient for child anxiety disorders as most children who would benefit do not access treatment [1-4]. Internet-based cognitive behavioral therapy (iCBT) for child anxiety is effective and acceptable within research settings [5,6]; however, user engagement, uptake, and adherence remain as challenges within routine clinical practice [7-9].

Involving the intended users in the development process and following a user-centered approach is crucial to maximize service user engagement and adoption of internet-based interventions within routine clinical practice [10,11]. User-centered design is different from the traditional approach of expert-led intervention development *for* users and embraces *active* collaboration *with* users to ensure that the digital solution is usable and meets their needs and preferences [11,12]. Key elements of user-centered design include (1) identifying user needs, preferences, and expectations for the digital solution; (2) actively involving users in the design and prototyping; and (3) conducting usability testing on a working prototype to provide feedback and identify technical and esthetic issues that may affect user satisfaction [10]. User-centered design can improve (1) design quality [13], (2) user adherence [14], (3) usability [15], (4) efficacy and sustainability [16], and (5) stakeholder acceptance and adoption at the system and organizational levels [17]. Recent iCBT development guidelines advocate active collaboration with users [10,18,19], and funding agencies increasingly demand stakeholder involvement.

Despite a clear rationale for user-centered design, few existing mental health digital interventions for young people have incorporated these principles within their development process. However, the number of digital interventions designed with young people is growing, with recent examples for depression [20,21], positive mental health [22], and recovery from mild traumatic brain injury [23]. Others have conducted usability testing of digital interventions for self-harm [24,25], depression [26], positive mental health [27], and recovery from pediatric cancer [28]. To the best of our knowledge, no existing iCBT interventions for child anxiety disorders actively involved users in the design process and only one intervention (*Breathe*, which is focused on adolescent anxiety) conducted usability testing [29]. This study highlighted the importance of involving both young people and clinicians in providing feedback in the iterative usability testing process, and the final iteration of *Breathe* showed improved usability and acceptance following this process.

### Aims

The aims of this study are to (1) collaborate with users to design an internet-based treatment for child anxiety disorders (phase 1) and (2) test the usability of this internet-based treatment (phase 2). Existing iCBT interventions for child anxiety disorders involve providing direct support to children or adolescents, via internet-based sessions or modules [5]. However, face-to-face CBT for child anxiety disorders can be delivered in a brief format directly to parents alone [30,31]. Therefore, we set out to design an internet-based treatment based upon a face-to-face parent-led CBT for child anxiety disorders that is effective compared with waitlist [30], is cost-effective compared with another brief psychological intervention (Solution-Focused Therapy [31]), is acceptable and feasible for use within routine clinical practice [32], and is now widely used in early intervention services in the United Kingdom [33]. This brief face-to-face treatment for child anxiety disorders involves the parent reading chapters of the accompanying treatment book [34] and meeting with a therapist for approximately 5 hours of support over approximately 8 weeks, to help apply the CBT strategies with their child and problem solve any difficulties [35]. Although the child is not seen by the therapist during treatment, the parent is encouraged to work through the CBT techniques collaboratively with their child and involve them throughout the process.

Before the design phase, we developed an initial overview plan for the internet-based version of this treatment, in consultation with stakeholders via our research clinic patient and public involvement group. This initial plan specifies that the internet-based version of the treatment would involve a parent website, case management system for clinicians, and a mobile game app for children that could be downloaded onto a smartphone or tablet. The intention is that parents would work through modules containing treatment material adapted from the book, with videos and animations to help demonstrate the CBT strategies. Clinicians would use the case management system to view the parents’ responses provided on the parent website and to release the next treatment module after a 20-minute telephone review therapist session. The plan to include a mobile game app for children was developed in response to feedback from our patient and public involvement group that it is important to incorporate opportunities to involve and motivate children in the treatment process. The intention of the game app for children is to help motivate the child in engaging in the treatment strategies such as facing their fears (graded exposure).

## Phase 1: User-Centered Design

### Methods

#### Participants

A total of 7 parents (n=6, 86% mothers and n=1, 14% father) and 4 children (n=2, 50% boys and n=2, 50% girls) aged 9-12 years were recruited. Of the 7 parents and 4 children, 5 (71%) parents and 4 (100%) children had recently received the brief face-to-face, parent-led CBT treatment and were recruited from a local child and adolescent mental health service, and 2 (29%) parents had received the face-to-face treatment several years ago as part of a research trial [30]. A total of 11 clinicians (n=7, 64% women and n=4, 36% men) who had experience of delivering the face-to-face treatment were recruited from 2 local child and adolescent mental health services. Sample characteristics are provided in Table 1.

**Table 1:** Demographic characteristics of phase 1 and phase 2 participants.

Participant group and characteristic					Phase 1			Phase 2
**Parents, n**					7			7^a^
	Age (years), mean (SD)			42.43 (5.68)			45.86 (9.84)	
	Sex (women), n (%)			6 (86)			6 (86)	
	**Relationship with child, n (%)**							
		Mother	6 (86)			6 (86)		
		Father	1 (14)			1 (14)		
	**Ethnicity, n (%)**							
		White British	6 (86)			5 (71)		
		White Irish	0 (0)			1 (14)		
		Other White background	1 (14)			1 (14)		
	**Highest level of education, n (%)**							
		School completion	0 (0)			1 (14)		
		Further education (eg, college and vocational course)	4 (57)			3 (43)		
		Higher education (undergraduate degree)	2 (29)			1 (14)		
		Postgraduate qualification	1 (14)			2 (29)		
	**Employment status, n (%)**							
		Unemployed	1 (14)			0 (0)		
		Part-time work	2 (29)			2 (29)		
		Full-time work	4 (57)			3 (43)		
		Retired	0 (0)			2 (29)		
**Children, n**					4			4^b^
	Age (years), mean (SD)			10.25 (1.26)			9.50 (0.58)	
	Sex (women), n (%)			2 (50)			2 (50)	
	**Ethnicity, n (%)**							
		White British	3 (75)			3 (75)		
		Mixed: White and Black African	1 (25)			1 (25)		
**Clinicians, n**					11			8^c^
	Age (years), mean (SD)			40.36 (9.28)			41.88 (9.42)	
	Sex (women), n (%)			7 (64)			4 (50)	
	**Ethnicity, n (%)**							
		White British	10 (91)			6 (75)		
		Other White background	1 (9)			2 (25)		
	**Professional background, n (%)**							
		Clinical psychologist	7 (64)			6 (75)		
		CBT^d^ therapist	1 (9)			1 (13)		
		Assistant psychologist	1 (9)			0 (0)		
		Social worker	1 (9)			1 (13)		
		Child and adolescent psychiatrist	1 (9)			0 (0)		

^a^A total of 5 parents participated in both phase 1 and phase 2.

^b^A total of 3 children participated in both phase 1 and phase 2.

^c^A total of 6 clinicians participated in both phase 1 and phase 2.

^d^CBT: cognitive behavioral therapy.

#### Measures

Participants reported on their technology use using a customized questionnaire. We collected feedback from parents and clinicians on each screen of initial mock-ups of the parent treatment website and clinician case management website, respectively, using an adapted version of the Program Content and Usability Questionnaire (PCUQ). Shortened versions of the PCUQ were administered to children to obtain feedback on various game visuals (character type, environment, and style), existing mobile game app types (story-led games, minigames, and virtual toys), and pen-and-paper mock-ups of the game (see Multimedia Appendix 1 [29,36,37] for full measure details).

#### Procedure

In all, 3 workshops were conducted separately for each user group (parents, children, and clinicians) approximately 1 month apart. Participants were financially compensated for their time and any travel expenses. Members of the university research team and the website development company conducted the workshops at varied times to facilitate participation.

A breakdown of the content for each of the 9 workshops is presented in Table 2. The workshops for each user group followed an iterative process, whereby feedback from workshop 1 fed into workshop 2 and so forth. In workshop 1, participants provided written consent and completed the demographic (parents and clinicians only) and technology use questionnaire.

**Table 2:** Phase 1 workshop content.

Participant group	Workshop 1	Workshop 2	Workshop 3
Parents	Introductions and aim for the workshop Warm-up exercise Explanation of the project Discussion on (1) the treatment itself, (2) how they would use internet-based version, and (3) therapist support: what and how MoSCoW^a^ card-sorting task for functions Rapid visual prototyping of top 1 or 2 functions	Introductions and aim for the workshop Warm-up exercise Talk through user journey using initial prototype Feedback (PCUQ^b^) on initial prototype Group discussion on initial prototype Rapid visual prototyping of areas identified for improvement	Introductions and aim for the workshop Warm-up exercise Talk through user journey using revised prototype Feedback (PCUQ) on revised prototype Group discussion on revised prototype Read through and discussion of excerpt of treatment content Feedback on plans for child app
Children	Introductions and design own name badge Explanation of the project Warm-up game Draw favorite superhero or character Discussion of (1) what the character would be scared of, (2) how they would face fears, (3) where they would live, and (4) rewards for facing fears	Introductions and design own name badge Recap of the project Warm-up game Feedback (PCUQ) on game visuals: (1) character type, (2) environment, and (3) style Feedback (PCUQ) on existing game types: (1) story-led games, (2) minigames, and (3) virtual toy	Introductions and design own name badge Recap of the project Talk through concept of the game and initial designs Feedback (PCUQ) on mock-up game: (1) character, (2) home screen, (3) dress-up game, (4) challenges, and (5) minigames
Clinicians	Introductions and aim for the workshop Warm-up exercise Explanation of the project Discussion on (1) concerns and positive aspects about providing the treatment via the internet, (2) issues in the face-to-face treatment, (3) clinician involvement in the internet-based treatment, and (4) digital tools currently used MoSCoW card-sorting task for functions Rapid visual prototyping of top 1 or 2 functions	Introductions and aim for the workshop Warm-up exercise Talk through user journey using initial prototype Feedback (PCUQ) on initial prototype Group discussion on initial prototype Rapid visual prototyping of areas identified for improvement	Introductions and aim for the workshop Warm-up exercise Talk through user journey using revised prototype Feedback (PCUQ) on revised prototype Group discussion on revised prototype Read through and discussion of excerpt of treatment content Feedback on plans for child app

^a^MoSCoW: Must have, Should Have, Could Have, Won’t Have.

^b^PCUQ: Program Content and Usability Questionnaire.

The aims of workshop 1 for parents and clinicians were to (1) present the overall concept of the internet-based intervention, (2) explore participants’ wants and needs, and (3) generate ideas about what the internet-based intervention might look like and the functionality it may have. Parents focused on the parent treatment website and clinicians focused on the clinician website. The MoSCoW approach (Must have, Should have, Could have, Won’t have) [38] was used to gain insight into what the priority of functions should be and what functions were not wanted by users. Participants quickly sketched their ideas for what key functions would look like using rapid visual prototyping [39]. Early prototypes of the websites were created from the learnings from workshop 1 and presented to the participants in workshop 2 to gain their feedback through discussion and the PCUQ. These early prototypes were interactive screens that could be navigated to represent the intended user experience. This purported to evaluate what the participants liked and what refinements were needed at a very early stage of development. Refined prototypes were presented in workshop 3 and participant feedback was collected through discussion and the PCUQ. The learnings from workshop 3 subsequently informed the development of the working prototype that was used in the usability testing (phase 2).

The aim of workshop 1 for children was to gain an understanding of the kind of character the game should have and how the game could help them face their fears. In workshop 2, children were presented with various game visuals in a colorful booklet and they played different types of existing games on a tablet. Children completed the PCUQ to obtain feedback on what children wanted the game to look like and the type of game they preferred. Pen-and-paper mock-ups of the game were created based on this information and presented to children in workshop 3. Children provided their feedback on the mock-ups through discussion and the PCUQ. This informed the development of a working prototype of the game that was used in the usability testing (phase 2).

During the workshops, detailed field notes were taken to capture the discussion, and visual activities were photographed. Following each workshop, members of the research team and website development company met to collate and summarize feedback from the discussion, activities, and the PCUQ.

#### Data Analysis

Phase 1 involved gathering information to inform the subsequent development of the intervention, and therefore, no formal statistical analysis was conducted. Key learnings and descriptive statistics for the PCUQ are presented below. The outcomes of phase 1 were a description of the needs and wants of the intended user groups for Online Support and Intervention for child anxiety (OSI) and confirmation that these have been met in the early prototypes.

#### Ethics Approval

The study was approved by the University of Reading Research Ethics Committee (16/48) and the National Health Service South East Coast–Surrey Research Ethics Committee (16/LO/1598).

### Results

#### Technology Use

As shown in Table 3, all the parents and clinicians had regular access to the internet and a PC or laptop, and most of them had regular access to a smartphone. Parents and clinicians reported feeling confident in using these technologies and endorsed that they liked using them. Most of the clinicians had no experience with delivering internet-based psychological therapies. Children reported regular use of the internet and tablets, with some use of PCs or laptops and smartphones. All children rated themselves as confident in using and liking these technologies.

**Table 3:** Technology use of participants in phase 1 and phase 2.

Participant group and variables				Phase 1		Phase 2
**Parents, n**				7		7
	**Regular access to the device, n (%)**					
		PC or laptop	7 (100)		7 (100)	
		Internet	7 (100)		7 (100)	
		Smartphone	6 (86)		6 (86)	
		Tablet	5 (71)		5 (71)	
		None of the above	0 (0)		0 (0)	
	**Confidence^a^ in using the device, mean (SD)**					
		PC or laptop	4.14 (0.69)		4.29 (0.76)	
		Internet	4.14 (0.69)		4.29 (0.76)	
		Smartphone	4.33 (0.52)		3.86 (1.35)	
		Tablet	4.33 (0.52)		4 (1.41)	
	Liking^b^ for using these technologies, mean (SD)		4.14 (0.69)		4.29 (0.76)	
**Children, n**				3^c^		4
	**Frequency^d^ of using the device, mean (SD)**					
		PC or laptop	3.33 (0.58)		3.25 (0.50)	
		Internet	4 (0)		4 (0)	
		Smartphone	3 (1)		2.75 (0.96)	
		Tablet	4 (1)		4 (0.82)	
	**Confidence^a^ in using the device, mean (SD)**					
		PC or laptop	4.33 (0.58)		4.50 (0.58)	
		Internet	4.67 (0.58)		4.75 (0.50)	
		Smartphone	4.67 (0.58)		4.75 (0.50)	
		Tablet	4.67 (0.58)		4.75 (0.50)	
	Liking^b^ for using these technologies, mean (SD)		5 (0)		4.75 (0.50)	
**Clinicians, n**				11		8
	**Regular access to the device, n (%)**					
		PC or laptop	11 (100)		8 (100)	
		Internet	11 (100)		8 (100)	
		Smartphone	9 (82)		7 (88)	
		Tablet	6 (55)		4 (50)	
		None of the above	0 (0)		0 (0)	
	**Confidence^a^ in using the device, mean (SD)**					
		PC or laptop	4.45 (0.52)		4.25 (0.46)	
		Internet	4.45 (0.52)		4.25 (0.46)	
		Smartphone	3.91 (1.04)		3.88 (0.99)	
		Tablet	3.73 (1.10)		3.57 (0.98)	
	Liking^b^ for using these technologies, mean (SD)		4 (0.63)		4.13 (0.64)	
	**Experience of delivering web-based psychological therapies, n (%)**					
		No experience	7 (64)		6 (75)	
		A little experience	3 (27)		2 (25)	
		Some experience	0 (0)		0 (0)	
		Quite a lot of experience	0 (0)		0 (0)	
		Lots of experience	1 (9)		0 (0)	

^a^Rated on a 5-point Likert scale, with higher scores indicating greater confidence.

^b^Rated on a 5-point Likert scale, with higher scores indicating greater liking.

^c^Data missing for 1 participant.

^d^Rated on a 5-point Likert scale, with higher scores indicating more frequent use.

#### Workshops

The key learnings from workshop 1 are presented in Textbox 1. Overall, parents and clinicians were positive about the idea of creating an internet-based version of the face-to-face treatment, and children were excited about a game that could help them face their fears.

The functionality proposed for the parent and clinician OSI websites was largely welcomed, with the exception of secure videoconferencing for the therapy sessions. Although clinicians considered this as a *must have*, they had concerns about the technology failing and the implications this could have partway through a therapy session. In contrast, parents did not want the therapy session to be held over a secure videoconferencing system and preferred telephone contact. In response to this feedback from parents, secure videoconferencing was not included in the prototypes presented in subsequent workshops.

Both parents and clinicians suggested additional functionality that they wanted for OSI. For parents, this included a *favorites* section where parents could bookmark key parts of the treatment modules and a written summary of the therapy session. Clinicians suggested that a *welcome* module would be helpful to orientate parents to the treatment approach.

The children wanted to be able to personalize the character in the game through customizable elements such as hair, clothes, and color. They thought the game could help them face their fears if they could earn rewards through it for facing their fears on their *step plan* (exposure ladder).

Initial prototypes of the parent and clinician OSI websites were presented to parents and clinicians, respectively, in the subsequent design workshops (workshop 2 and workshop 3). Overall, the PCUQ results showed that parents and clinicians were positive about the proposed design and functionality (Table 4). Feedback provided during workshop 2 helped to further improve the intervention, as shown by the higher PCUQ ratings during workshop 3.

The main finding from the children’s design workshop 2 was that they were largely positive about the game visual options, with a slight preference for a mountain setting and cartoon style for the game app over alternative designs (Multimedia Appendix 2). They liked the concept of having more than one game type to choose from, and their favorite minigame type was *bottle-flipping* [40]. In workshop 3, children were presented with pen-and-paper mock-ups of the game. Children rated all aspects of the game dynamics specified in the PCUQ (game character, home screen, dress-up game, game selection screen, and challenges) as highly liked and easy to use (Multimedia Appendix 3). All the game play options presented were also rated as highly liked and easy to use. Overall, the children agreed that the game developer had understood what they wanted and few changes were needed. On combining feedback from workshops 1 and 2, game option 1 (monster flipping) was selected as the primary game.

**Key learnings from workshop 1.**

**Key learnings from parents:**

- Therapy session to be conducted via telephone and not via secure videoconferencing.
- Include a *favorites* section.
- Clinician should provide a written summary of the therapy session.

**Key learnings from children:**

- Ability to personalize the character in the game.
- Earn rewards through the game and step plan.

**Key learnings from clinicians:**

- Not include an instant messaging service with the parent.
- Conduct the therapy session over secure videoconferencing built into system but concerned about technical issues.
- Not include one-way messages of encouragement to the child app.
- Include a *welcome* module to orientate parents to the website and clarify expectations of the treatment approach.

**Table 4:** Phase 1 parent and clinician feedback on initial mock-ups of Online Support and Intervention for child anxiety.

PCUQ^a^ item^b^	Parents, mean (SD)		Clinicians, mean (SD)	
	Iteration 1	Iteration 2	Iteration 1	Iteration 2
It looks easy to use	4.78 (0.32)	4.86 (0.19)	4.36 (0.43)	4.25 (0.32)
It looks easy to navigate	4.75 (0.37)	4.89 (0.15)	4.21 (0.65)	4.37 (0.23)
The words are clear and easy to understand	4.72 (0.35)	4.94 (0.15)	4.23 (0.47)	4.37 (0.45)
This screen has the right amount of information	4.44 (0.57)	4.86 (0.26)	4.04 (0.49)	4.30 (0.46)
This page is visually pleasing to me	4.37 (0.51)	4.60 (0.55)	3.71 (0.64)	4.13 (0.45)
It looks clear what to do next	4.37 (0.71)	4.80 (0.34)	3.64 (0.48)	3.92 (0.23)
This page looks user-friendly	4.45 (0.56)	4.85 (0.20)	3.93 (0.47)	4.17 (0.49)
Based on this page, I would return to this website	4.61 (0.34)	4.83 (0.18)	3.93 (0.62)	4.02 (0.60)
The tone of the material is sensitive for parents seeking help for their child’s anxiety	4.64 (0.45)	4.86 (0.18)	—^c^	—
The material is relevant for parents seeking help for their child’s anxiety	4.61 (0.27)	4.85 (0.19)	—	—
This page meets my needs for an online treatment program	4.64 (0.30)	4.77 (0.29)	3.96 (0.34)	4.18 (0.42)
The page meets what I would want from an online treatment program	4.56 (0.39)	4.82 (0.20)	3.96 (0.30)	4.17 (0.46)
This page meets my expectations as discussed in the workshop	4.57 (0.39)	4.86 (0.16)	3.79 (0.47)	4.05 (0.63)

^a^PCUQ: Program Content and Usability Questionnaire.

^b^Items were scored on a scale of 1 (strongly disagree) to 5 (strongly agree).

^c^Questionnaire item was not relevant for clinicians to answer.

## Phase 2: Usability Testing

### Methods

#### Participants

A total of 7 parents (n=6, 86% mothers and n=1, 14% father), 4 children (n=2, 50% girls and n=2, 50% boys), and 8 clinicians (n=4, 50% women and n=4, 50% men) participated in phase 2. Sample characteristics are presented in Table 1. In all, 71% (5/7) of the parents (all mothers), 75% (3/4) of the children, and 75% (6/8) of the clinicians participated in both phase 1 and phase 2. Participants were recruited using the same approach as phase 1.

#### Measures

Participants completed the technology use and PCUQ questionnaires, as described above and in Multimedia Appendix 1 [29,36,37]. Participants completed the PCUQ in reference to OSI (parents and clinicians) and the game (children) as a whole, rather than specific to each function of the website as in phase 1.

##### Usability Error Log

Usability errors during use of the parent and clinician websites were logged according to severity metrics common in usability testing [41,42]: (1) level-1 errors refer to cosmetic or minor factors such as typos, legibility, or esthetic preference; (2) level-2 errors are moderate issues; for example, inability to navigate successfully through the site; and (3) level-3 errors are critical or severe issues, such as technical issues that prevents the user from using a function of the site. The frequency of each error severity level was summed for each iteration.

##### Semistructured Interviews

Semistructured interviews were used to gather further information about the acceptability and ease of use of the website and game. Questions were open and nonspecific to encourage unprompted reports of what was most relevant. Specifically, parents and clinicians were asked the following: (1) What did you think of the website overall? (2) What did you find easy to use? (3) What did you find difficult to use? (4) What did you find easy to understand? (5) What did you find difficult to understand? (6) What did you like about it? (7) What did you dislike about it? (8) What do you think about how long it took to complete a module (parents only)? (9) What would you like to change about the website? (10) What would you like to add to the website? (11) What would you like to remove? (12) What do you think about the colors used? and (13) Is there anything else you would like to say about the website? At this point, we suggested the name, OSI, to parents and clinicians and asked for their feedback. For each element of the game (ie, home screen, monster flip game, and monster dress-up game) children were asked the following: (1) What did you like about the game? (2) What did you dislike about the game? (3) What did you find easy to use? (4) What did you find hard to use? and (5) What would you like to change about the game? Children were also asked what they thought about the sounds and music used in the game. Interviews were audio-recorded and transcribed verbatim for analysis.

#### Procedure

Participants attended individual usability testing sessions at the University of Reading at a time convenient to them. Parents and clinicians were shown the website relevant to them and asked to *think aloud* (ie, provide continuous commentary) [43] while carrying out a list of tasks (eg, parents were asked to work through the module and bookmark a page and clinicians were asked to book an appointment for a therapy session) and were provided time to explore the website by themselves. A researcher was present to record the frequency and detail of usability errors using the usability error log. Both the participant and the screen were video-recorded, in case further clarification was required after the testing process. Then, parents and clinicians completed the PCUQ and semistructured interview. Usability testing sessions for the children followed a similar format. Children were asked to explore the game and say out loud what they thought of it as they played it (ie, the *think aloud* method [43]). A researcher was present to record observational notes to obtain a general idea of how children proceeded through the game. Then, the children completed the child PCUQ and semistructured interview.

The research team collated and summarized the feedback on each aspect of the website and the game from the first round of usability testing. Then, the website development company revised the website and the game in response to the user feedback. Then, the second iterations were presented to participants in a second round of usability testing, following the same procedures outlined above. Additional functions were also included in the subsequent iterations of OSI. Feedback from the second round of usability testing was used for further iteration, and a third (final) round of usability testing was conducted as described above. Feedback from the final round of usability testing was used to finalize the website and the game.

#### Data Analysis

Descriptive statistics were used to analyze the PCUQ responses and the frequency of usability errors for each iteration of the website for both parents and clinicians. Brief summaries of the semistructured interviews were used to identify immediate feedback on each iteration of the website and game that could be used to further iterate OSI. Then, a content-analysis approach was used to analyze interview transcripts across all 3 interactions to capture more detailed insights into the acceptability and ease of use of the website and game. Responses were assigned codes according to whether the comment reflected positive feedback or negative feedback or suggested improvement and the aspect of the design or functionality of the website or game discussed. Then, the codes were organized according to these categories to identify key themes and patterns across the 3 iterations for each set of interviews (parents’, clinicians’, and children’s interviews). The analysis took into account both frequency and severity of usability issues discussed in the interviews. A summary of the key themes along with example quotations from the interviews are presented below.

### Results

#### Technology Use

Parents and clinicians in phase 2 had regular access to the internet and a PC or laptop, and most of them also had regular access to a smartphone (Table 3). Most of the participants reported feeling confident with and liking these technologies. Most clinicians had no experience of delivering internet-based psychological therapies. Children reported regular use of the internet and tablets, with some use of PCs or laptops and smartphones. All children rated themselves as confident in using and liking these technologies.

#### Usability Testing Sessions

Feedback from parents and clinicians on working prototypes was predominantly positive across the 3 iterations, with mean scores on the PCUQ largely indicating *agree* or *strongly agree* (Multimedia Appendix 4). For most items on the PCUQ, ratings increased across the iterations, suggesting that usability had improved with the changes made to each iteration. Scores for “it is always clear what to do next” stayed within *neutral* to *agree* across iterations for clinicians. Children reported a similar trend for sustained or improved usability scores on the PCUQ for the majority of items (Multimedia Appendix 5); however, some items (“each screen has the right amount of information,” “it is easy to use the home screen,” and “each screen loaded quickly”) showed a slight decline in scores at iteration 3.

**Figure 1:**
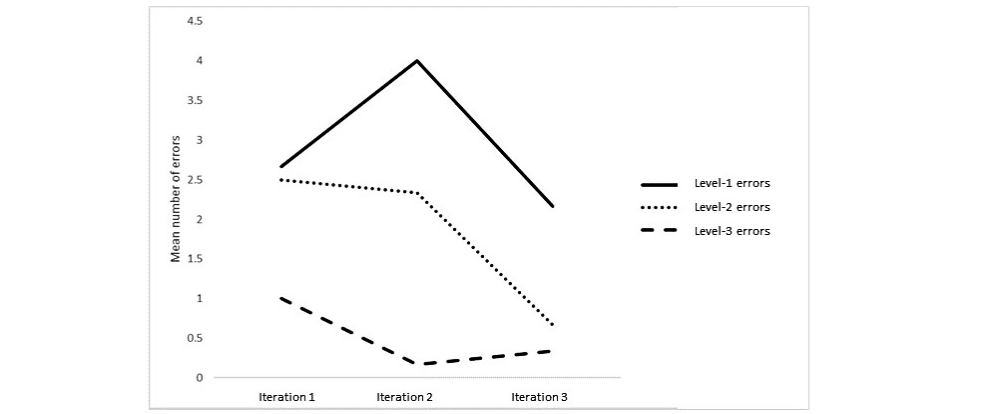
Mean number of usability error types for parents across the iterations of Online Support and Intervention for child anxiety.

**Figure 2:**
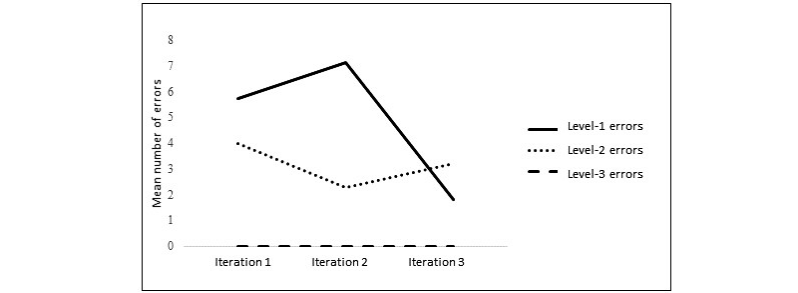
Mean number of usability error types for clinicians across the iterations of Online Support and Intervention for child anxiety.

Usability errors for parents and clinicians followed a similar pattern across the iterations (Figures 1 and 2). Overall, there were fewer level-3 errors (critical technical issues) than level-2 errors (moderate issues) or level-1 errors (minor or cosmetic issues) for all iterations. Level-3 errors stayed at a low level for parents across all iterations and none occurred for clinicians. Level-1 and level-2 errors tended to reduce across the iterations, with the exception of level-2 errors for clinicians that showed a slight increase at iteration 3, which may be owing to the introduction of new features to the case management system at this stage.

#### Semistructured Interviews

Results from the semistructured interviews reflected the findings from the PCUQ and usability errors.

##### Parents

Parents were positive about the overall structure of the website, how the treatment material was presented, and the time required to complete the modules:

What I liked about it is it’s something I can go back to in my own time when I can’t remember exactly how to do something, and it’s easier to access and use because it’s in nice bite-size chunks, rather than having to flip through the book or my notes from the course.

Parent 7; iteration 3

I think the information is not too much, straight to the point and also, the fact that you’re...it’s not just words there are videos, so yes I think that’s important. I think it’s not long, I think it’s a good time.

Parent 2; iteration 2

Parents were positive about the website—in particular, that it was easy to use and the materials were accessible:

I like the fact that you can at any point stop and go to any other section that you wish to, you don’t have to complete that whole section, you can go backwards and forwards as much as you like and it’s very quick and easy to do that.

Parent 4; iteration 1

Well actually, some of the functionality I thought might have been difficult, in other words, adding items to the agenda, moving items around, reviewing bookmarks that I’ve added, adding in comments and all those sort of things were good.

Parent 7; iteration 2

Of note, there was only 1 comment across all the parent interviews relating to technical issues*:*

The bit that was a bit frustrating was the, where it sometimes jumped back to the beginning which I think was a Mac-ism.

Parent 7; iteration 2

The look and feel of the website were also viewed positively; the OSI name, layout, and language used contributed to this:

I like that, OSI. I think it’s simple, it’s easy to understand and easy to remember. OSI. OSI for this, OSI for that, yeah.

Parent 2; iteration 1

It’s pretty easy to use, it’s well laid out so you know where everything is, the menu’s quite easy to understand where all the things are, and so yeah. It’s easy to navigate.

Parent 2; iteration 2

I think that the text is really good, I think that’s gonna help it to be as straightforward and painless as possible.

Parent 5; iteration 3

Parents provided some useful feedback on the colors; in the first iteration, a parent noted that, “the blue and white is quite clinical. If I was to pick a colour I’d pick green, because it’s restful” [Parent 1; iteration 1]. This feedback was used to develop the website, and at the next iteration, the same parent spoke positively about the revised colors:

It’s definitely more pleasing to the eye now, with the colours that have been changed from the previous session that I did, the blue just didn’t work for me...it just felt too medical and I think when you’re in that whole, dealing with the whole anxiety thing it just dominates your whole world, so to be on it when that child is in bed or at school or whatever, to then still be very medical...the green is a lot more relaxing and kind of gently supportive really I suppose.

Parent 1; iteration 2

Other useful feedback at iteration 2 were related to the animation and videos:

I didn’t like the animation. It was too long. And I didn’t like the music. With it. Erm, I would have preferred the real people in that bit.

Parent 4; iteration 2

Well apart from the video, I couldn’t understand the person talking, and I think subtitles or the optional subtitles might be useful.

Parent 2; iteration 2

Changes made at iteration 3 were noticed and elicited positive feedback:

I did really dislike the talking heads the last time, I much preferred the true person talking...I much preferred the video, the talking. The real person talking.

Parent 4; iteration 3

Parents talked about a key element of the treatment—the step plan, which they were keen to optimize, and they recognized it as something they would want to revisit:

I think I had a bit of a challenge with the step plan bit, I think that needs to be a bit finetuned and changing the names of it, and going to steps, but otherwise it’s pretty straightforward.

Parent 2; iteration 1

I think if you were going back and you wanted to listen to it again just to pick out the main points, you’d be thinking ugh it’s a bit...I think the look was fine, I just, if it was more concise.

Parent 3; iteration 2

This feedback was responded to, and at iteration 3, parents reported more favorably about the step plan video:

I much prefer the new step plan [video].

Parent 3; iteration 3

##### Children

Overall, children were positive about the game app and found it easy to use. For the monster flip minigame, all of them found the game accessible (eg, “it was easy to use. I like how it explained what to do before, so you knew what to do” [Child 3; iteration 2]), but provided enough challenge to be interesting (eg, “think the hardest part of the game is judging where he’s gonna bounce” [Child 4; iteration 2]). As expected, some children found it harder than others to play; a child suggested that “there could be higher levels” [Child 1; iteration 3], whereas another child said that “the extreme one was too hard” [Child 3; iteration 3]; however, they still liked the game overall (eg, “it was a good background. I like how it like, showed where you were at the top” [Child 3; iteration 2]).

Regarding the dress-up game, all the children liked it, and all of them said that there was nothing they disliked about it:

I found the buying was easy to use...I like the clothes shop, because it was super easy.

Child 4; iteration 2

A child talked about the functionality of the dress-up game in terms of not knowing how to control the activity:

There was one thing, when I bought some of the clothes, I didn’t realise I had to drag it on, I just tapped it and I thought it would just go on.

Child 2; iteration 2

However, the other children found it easy to use. Although this was not a universal problem, the game instructions were refined to address this issue.

Children liked the home screen and commented on particular details:

I like the view out the window.

Child 3; iteration 2

I like the ability that you could drag the log to the fire and that you could get a newspaper which would entertain him, and I liked the design of the house, and the buttons are easy to use.

Child 2; iteration 3

They asked for more colors to be used, but only in response to a specific question about whether they disliked anything about the home screen:

Maybe like, change the colour scheme a bit because it was pretty much all blue, maybe you could paint the house? Or like I said maybe get a new house.

Child 2; iteration 3

Others commented positively on the colors:

I liked how you could like, go to whichever place you wanted to, and I like the colour of it.

Child 3; iteration 2

Feedback on the initial iteration included requests for more options for customizing the character:

I don’t like the fact that you can’t change him, you can’t choose his name, you can’t choose his colour, you can’t choose what he looks like.

Child 4; iteration 1

This was addressed in later iterations to the game with the addition of the dress-up game and the random name generator.

##### Clinicians

Overall, clinicians responded positively to the OSI case management system and acknowledged that any issues they had while using OSI may be because they were a novice user:

It was good, I liked it. It’s quite easy to kind of navigate, it’s quite obvious where to go for things, and when you’re actioning something it’s quite obvious what you need to do next. Like that it’s not too cluttered, it’s got kind of just what you need on there.

Clinician 8; iteration 1

The only thing I’m unsure of is the progress, the notes bit where you take like progress notes, but that’s just because I haven’t used the system much, after like one week I’d be, I’d know exactly where to go.

Clinician 7; iteration 3

Clinicians talked about specific key features of the website, including the calendar feature, the routine outcome measures (ROMs), and the notes function and how risk is managed within OSI (eg, “the week view would be useful. I think it would be helpful to have a month view as well” [Clinician 7; iteration 1]), and later noticed changes to these features without prompting (eg, “I like the calendar view, cos I think that’s changed from before” [Clinician 8; iteration 2]).

Clinicians were particularly positive about the ROMs:

ROMs bit was the easiest bit, and thinking about new clinicians coming in and being able to see the ROMs all on screen, presented in that way, showing the clinically significant change, whether the scores are above or below the cutoff line, the subscales mapped out with the dates, like I’m struggling to think of how you could display that in an easier way...so that was a nice bit I think. I think for new practitioners, will demystify the process of ROMs.

Clinician 4; iteration 2

Some clinicians had suggestions for improvements, but the suggested changes were not necessarily echoed by other clinicians. For example, a clinician found all elements acceptable:

...everything was labelled quite clearly, particularly bits in the client information are quite good, so the risks, adding notes and ROMs. I thought all of that information was really clear and easy to access.

Clinician 8; iteration 2

By the third iteration, clinicians struggled to identify any further changes they would like to suggest:

I don’t think there was anything I disliked. Nope. No nothing comes to mind I disliked...Well done. Am I allowed to say well done? Because having used so many unfriendly systems this is like a dream.

Clinician 6; iteration 3

Really good, really well structured and clean, and yeah it’s got the right information.

Clinician 4; iteration 3

#### Final Iteration of OSI

Multimedia Appendices 6-8 show screenshots of the final iteration of the OSI parent website, clinician case management system, and child game app, respectively [44]. Both the parent and clinician OSI websites can be accessed via internet browsers on a computer (desktop or laptop), smartphone, or tablet device.

The parent works through 7 modules (Multimedia Appendix 9) that are released weekly and take approximately 30 minutes to complete. Each module follows the same format with compulsory ROMs, module content, summary of module, homework for the week, module quiz (optional), and module feedback (optional). The ROMs include parent-report versions of the Revised Children’s Anxiety and Depression Scale (RCADS) [45], Child Anxiety Impact Scale (CAIS) [46], Child Outcome Rating Scale [47], Goal Based Outcomes [48], brief Spence Child Anxiety Scale [49], and the Session Rating Scale [47]. These ROMs are compatible with the Child and Young People Increasing Access to Psychological Therapies initiative in the United Kingdom [50]. In addition, the CAIS can detect meaningful clinical change in anxiety symptoms and interference [51], and the brief Spence Child Anxiety Scale works well as a brief measure of anxiety symptoms [49]. In line with Child and Young People Increasing Access to Psychological Therapies, all measures are collected weekly, except the CAIS and RCADS, the full versions of which are administered at the start and end of treatment, and a subscale is tracked weekly. Parents can immediately view the scores of the ROMs and information about how to interpret scores via the *your child’s progress* tab. Modules are interactive, with questions to answer and worksheets to complete. The treatment material is presented as easy-to-read text, with videos and animations to demonstrate the strategies and provide parent testimonials. Parents can bookmark module pages for quick reference and add notes throughout about their own reflections or experiences. Resources from within and outside OSI are found in a *resources* tab. After completing each module, the parent has a 20-minute telephone review session with their therapist. The telephone session aims to review how the parent applied the strategies with their child and problem solve any difficulties. Details on the therapy session appointment times are found in the *therapy sessions* tab, along with a written summary from the therapist for previous therapy sessions. Parents can add to the upcoming therapist session agenda throughout the relevant module and via the *therapy sessions* tab. They can also reorder items on the agenda to prioritize items that are important to them to discuss.

The OSI case management system allows the clinician to view the service users they are supporting on OSI via the *client list* tab. On clicking a child’s name, a detailed view of the child’s OSI case file is presented. This includes their personal details and all interactive elements of the parent site. Parent’s answers to questions and worksheets are shown under the *homework* tab for clinicians to review before the telephone review session. ROMs are scored and immediately available for review and download via the *ROMs* tab. Appointments for the telephone review session can be booked via the *appointments* tab, which also gives details of past and upcoming therapist sessions. Upcoming appointments can also be viewed via the *calendar* tab. Notes from the therapist sessions are found under *notes*, and therapists can also add additional progress notes here. Clinicians can view the OSI treatment material presented in a PDF format via the *view treatment* tab. Details on how to contact technical support and frequently asked questions on using OSI are provided for both parents and clinicians via the *help guides* tab. Clinicians are provided with a training video and clinician manual for the OSI case management system.

The child game app is called *Monster’s Journey: Facing Fears* and is available to download on smartphones or tablets via the Apple Store and Google Play Store. A registered parent OSI account is needed to access the game (for free). The game aims to help motivate the child to engage in the treatment strategies, and it is an optional part of the OSI treatment program. The game is introduced to families in module 3–*facing fears* and can be used as a reward option when the child faces their fear. Parents can reward their child with virtual coins via a parent portal in the game, which the child can use to unlock various features, such as different minigames, extra levels and modes of the games, and customization options. The game consists of a monster character who the child can name and personalize with different outfits. There are 3 minigames, which the child can choose to play. Each minigame has a range of modes and difficulty levels that can be unlocked using the virtual coins. Children can also earn a nominal number of virtual coins via *challenges* within the games. The home screen acts as a base for the monster and is where the child receives notifications of the reward of virtual coins and other gifts received during gameplay. The game is intended to be motivational and is not intended to be therapeutic in its own right. However, introductions to the minigame includes storylines about helping the monster to face its fears, and there are messages of encouragement to continue playing when the child is struggling with the gameplay and of positive reinforcement when they succeed. Parents are provided with guidance on how to introduce the game and manage screen time.

## Discussion

### Principal Findings

OSI was developed through a process of user-centered design and usability testing. The results presented here show that by adopting this approach, OSI meets the expectations and needs of the intended users (parents, children, and clinicians). The initial vision for OSI was confirmed and refined during the design workshops in phase 1 of development. This enabled participants to highlight what functions of OSI were important to them and how they would like these to look and work on the website and game. The initial positive ratings of the working prototypes of OSI presented during usability testing (phase 2) suggests that the active collaboration with users at the design stage was a successful and important part of the development process. The 3 cycles of usability testing allowed for further improvement of OSI and by the third iteration, participants reported high levels of satisfaction with how OSI looked, its ease of use, and the functions available.

It is noteworthy that clinicians reported that the OSI case management system was easy to use without any training. This suggests that OSI is intuitive to use; however, most of the clinicians participated in both phase 1 and phase 2 of the development process, and thus, had some degree of familiarity with OSI. Clinician ratings for “it is always clear what to do next” did not show the same degree of improvement across the iterations in phase 2 as the other items on the PCUQ. There was also a slight increase in the number of moderate issues (such as inability to successfully navigate through the site) at iteration 3. This could be owing to the introduction of additional features in the third iteration of the OSI case management system. Feedback gathered in the semistructured interviews highlighted that the clinicians felt that any difficulty in using potentially confusing elements of OSI (eg, the calendar) would resolve with regular use; however, changes were also made to address issues raised, and a clinician training package and clinician manual were subsequently developed to help clinicians to quickly adopt OSI as part of their routine clinical practice.

Parents consistently rated OSI as acceptable and easy to use throughout the design and usability process. This suggests that the initial workshop where parents decided on what features they wanted OSI to have and how they might look was crucial and successfully translated into acceptable and usable initial (phase 1) and working (phase 2) prototypes. Parents were overwhelmingly positive about OSI by the final iteration. It is encouraging that they reported it was simpler to use than they expected, the treatment material was appropriate in tone and presentation, and the time needed to work through modules was acceptable. This feedback is important given that engagement in and adherence to internet-based treatments is associated with treatment outcomes [52-54] but has been low in some previous studies [55-57]. Nevertheless, it will be important to continue to monitor and assess the acceptability of time needed to complete the modules and weekly questionnaires in future research.

Overall, the game app was positively endorsed by children. Some items to assess content and usability did not show a linear improvement across the iterations, and this is likely to be related to the addition of many new features across the usability testing period. Despite this, by iteration 3, children rated the game app as looking good and easy to use and understand, and they reported that they would use the game again. This is encouraging because the aim of the game is to help motivate the child to engage in the treatment strategies by acting as a reward mechanism; thus, a desire to play the game is essential. However, it is worth noting that the game is an optional part of treatment, and future research is needed to determine the extent of its use and whether it successfully motivates children to engage in the treatment strategies and ultimately enhances treatment outcomes. For example, future research could compare outcomes for children who use and do not use the game during treatment.

User-centered design and usability testing are considered as best practice for digital mental health innovation [10,18,19], and developers are encouraged to share their development process to help further understanding of how best to design digital mental health interventions [10]. We hope that the methods we used to develop OSI will provide a model that others can adapt for interventions for other target disorders or age groups. Although our study clearly illustrates the benefits of user-centered design and usability testing, it is worth acknowledging some of the key challenges associated with the process that will be relevant for others who are developing digital mental health interventions. The process was lengthy, spanning 10 months. It also required careful planning with the technology company that developed OSI to ensure that the timings of the research protocol were achievable and in line with the time taken to develop each iteration of OSI. Time pressures sometimes meant that some features were not available for testing until later stages in the usability testing process. Nonetheless, usability ratings improved over time, suggesting that the collaborative design of these features helped to ensure they were easy and enjoyable to use. It should also be acknowledged that time and budget constraints meant that not all suggestions for improvements could be implemented. Our approach was to respond to anything that caused significant usability issues for most participants or was critical for successful delivery of the treatment. The high acceptability and usability scores indicate that this did not adversely affect ease or enjoyment of use.

### Strengths and Limitations

This study has several strengths. Of primary importance, we adopted a bottom-up approach to the development. This allowed the participants to tell us what features they wanted and how they wanted them to look and work, which is the essence of participatory research [58]. By actively involving users at both the design stage *and* usability testing as part of the development process, we were able to not only understand the needs and wants for the website and game but also ensure that it met those expectations and was easy and enjoyable to use. Another strength is that we recruited participants who were familiar with receiving (parents and children) or delivering (clinicians) the treatment that OSI is based on. This enabled a rich discussion of the features that might be helpful in the internet-based translation of the treatment material and management of families receiving this treatment.

Despite the strengths, there are some limitations that should be acknowledged. Participants were predominantly from White British ethnic backgrounds, and parents were well educated and largely employed. The lack of diversity in sociodemographic characteristics means that we cannot be sure that OSI will be satisfactory and easy to use across other sociodemographic groups. Furthermore, the sample did not include those who were not regular internet users, and although 96% of UK adults (aged 16-54 years) report daily or almost daily use of the internet [59], there is marked variation in reasons for internet use and digital skills among the UK population [60]. However, efforts were made to mitigate these issues; for example, the text of the treatment material was considered *easy to read* in readability tests and a range of ethnic backgrounds was represented in the creation of the video and animations. Furthermore, each module has a feedback questionnaire, and thus, OSI can be further iterated based on user feedback from wider research and routine use. The minimum number of participants required for usability testing is often cited as 5 [61]; however, using larger sample sizes and ensuring that participants are representative of the target user group will increase the likelihood of detecting all usability issues [62]. We included >5 clinicians and parents who had experience of receiving and delivering child anxiety treatment, but only 4 children participated and all of them were aged between 9 and 12 years; thus, it is possible that our findings may not be applicable to younger children who might otherwise benefit from the treatment approach [30]. Although our sample size is consistent with previous usability testing of a child anxiety intervention [29], it is possible that there are usability problems that were not detected in this study. Finally, it is important to acknowledge that this study was conducted before the COVID-19 pandemic and associated physical distancing restrictions. It is possible that an increase in the use of digital tools during the pandemic may have changed user preferences and experiences of OSI, and it will be important to assess this through ongoing user feedback.

### Conclusions

In conclusion, taking an iterative approach to development through user-centered design and usability testing has resulted in an internet-based treatment for child anxiety (OSI) that appears to meet the needs and expectations of parents, children, and clinicians and is easy and enjoyable to use. Now, it is important to establish the effectiveness of OSI, and further research designed to evaluate OSI in clinical and community settings is in progress. Indeed, it is intended that OSI will continue to iterate further in response to feedback in these settings to ensure that it is helpful to families struggling with child anxiety problems and to clinicians who are supporting them.

## Appendix

Multimedia Appendix 1 Phase 1 measures.

Multimedia Appendix 2 Phase 1–children’s feedback on game visuals options.

Multimedia Appendix 3 Phase 1–children’s feedback on Online Support and Intervention for child anxiety game mock-ups.

Multimedia Appendix 4 Phase 2–parents’ and clinicians’ feedback on working prototypes of Online Support and Intervention for child anxiety.

Multimedia Appendix 5 Phase 2–children’s feedback on working prototypes of the game.

Multimedia Appendix 6 Screenshots of the Online Support and Intervention for child anxiety parent website.

Multimedia Appendix 7 Screenshots of the Online Support and Intervention for child anxiety case management system for clinicians.

Multimedia Appendix 8 Screenshots of the Online Support and Intervention for child anxiety child game app.

Multimedia Appendix 9 Online Support and Intervention for child anxiety treatment content.

## Abbreviations

CAIS: Child Anxiety Impact Scale
CBT: cognitive behavioral therapy
iCBT: internet-based cognitive behavioral therapy
NIHR: National Institute for Health Research
OSI: Online Support and Intervention for child anxiety
PCUQ: Program Content and Usability Questionnaire
RCADS: Revised Children’s Anxiety and Depression Scale
ROM: routine outcome measure
