# Supplementary material for: Online Support and Intervention for Child Anxiety (OSI): Development and Usability Testing
Source: JMIR Form Res. 2022 Apr 13;6(4):e29846. doi: 10.2196/29846 (PMC9047721; doi:10.2196/29846)
Supplement: Multimedia Appendix 1 [file formative_v6i4e29846_app1.docx]

**Multimedia Appendix 1. Phase 1 measures.**

**Technology use questionnaire**

Parents and clinicians were asked to report 1) what technologies (personal computer/laptop, internet, smartphone, tablet) they have regular access to at home and at work, 2) how confident they feel using each of these technologies (five-point likert scale ‘not at all confident’ to ‘very confident’) and how much they like using these technologies in general (five-point likert scale from ‘strongly dislike’ to ‘strongly like’). Children were asked 1) how often do they use various (personal computer/laptop, internet, smartphone, tablet) technologies (‘everyday’ to ‘never’), 2) how confident they feel using each of these technologies (five-point likert scale ‘not at all confident’ to ‘very confident’) and how much they like using these technologies in general (five-point likert scale from ‘strongly dislike’ to ‘strongly like’). Children were guided through the questionnaire by a member of the research team.

**Programme content and usability questionnaire (PCUQ**)

An adapted version of the PCUQ was used to collect feedback from parents and clinicians on each screen of initial mock-ups of the online treatment website and clinician portal website respectively. It was based on the PCUQ used by Wozney et al. [1] that was adapted from the System Usability Scale (SUS) [2] and the Standardised User Experience Percentile Rank Questionnaire (SUPR-Q) [3]. The PCUQ contained items that were applicable such as ‘It looks easy to navigate’ and ‘This page (screen) has the right amount of information’ but omitted items that were not relevant due to the limited functionality of the initial website mock-ups e.g. ‘The video segments loaded quickly’. Three additional items were included that were study-specific: ‘This page meets my needs for an online treatment programme’, ‘The page meets what I would want from an online treatment programme’ and ‘This page meets my expectations as discussed in the workshop’. Two items were omitted from the clinician PCUQ as they were specific to the parent website (‘The tone of the material is sensitive for parents seeking help for their child’s anxiety’ and ‘The material is relevant for parents seeking help for their child’s anxiety’). Response options for all PCUQ items were on a 5-point Likert scale ranging from 1 ‘strongly disagree’ to 5 ‘strongly agree’. Responses on each questionnaire item were averaged across all screens for each iteration for analysis.

Shortened versions of the PCUQ were administered to children to obtain feedback on various game visuals (character type, environment, style), existing mobile game app types (story-led, mini-games, virtual toy) and pen and paper mock-ups of the game. There were four items for each game visual: 1) How much do you like the look of this? (five point likert scale ranging from 1 to 5 ‘I hate it’ to ‘I love it’), 2) Have the people who made the game understood what you wanted? (three response options: ‘yes’, ‘some of it’ and ‘no’), 3) Would you like us to change this? (two response options: ‘yes’, ‘no’), 4) What would you like us to change? (free-response). These items plus an additional item of ‘How easy to use does this look? (five point likert scale ranging from 1 to 5 ‘really hard’ to ‘easy’) was used to collect feedback on the mock-up games. There were just two items for existing game types: 1) How much do you like the game? (5 point likert scale ranging from 1 to 5 ‘I hate it’ to ‘I love it’) and 2) What did you like or dislike about the game? (free response). Children were guided through the PCUQ by a member of the research team.

**References**

1. Wozney L, Baxter P, Newton AS. Usability evaluation with mental health professionals and young people to develop an Internet-based cognitive-behaviour therapy program for adolescents with anxiety disorders. BMC pediatrics. 2015;15(1):1-11. doi: 10.1186/s12887-015-0534-1.

2. Bangor A, Kortum P, Miller J. Determining what individual SUS scores mean: Adding an adjective rating scale. Journal of usability studies. 2009;4(3):114-23.

3. Sauro J. SUPR-Q: A Comprehensive Measure of the Quality of the Website User Experience. Journal of useability studies. 2015; 10(2): 68-86.
