# Supplementary material for: Online Support and Intervention for Child Anxiety (OSI): Development and Usability Testing
Source: JMIR Form Res. 2022 Apr 13;6(4):e29846. doi: 10.2196/29846 (PMC9047721; doi:10.2196/29846)
Supplement: Multimedia Appendix 2 [file formative_v6i4e29846_app2.docx]

Multimedia Appendix 2. Phase 1 children’s feedback on game visuals options.

| Aspect of game visual | Like^1^ (mean, SD) | Developer understood what wanted^2^ (% yes (n)) | % (n) want it changed |
| --- | --- | --- | --- |
|  |  |  |  |
| *Character type* |  |  |  |
| - Weird | 4.25 (.50) | 66.7 (2) | 25 (1) |
| - Animal | 5 (0) | 66.7 (2) | 25 (1) |
| - Monster | 4 (0) | 66.7 (2) | 25 (1) |
| *Environment* |  |  |  |
| - Forest/jungle | 4.5 (.58) | 66.7 (2) | 50 (2) |
| - Mountains | 4.75 (.50) | 66.7 (2) | 0 (0) |
| - Space | 4.50 (.58) | 100 (3) | 50 (2) |
| - Under the sea | 4.75 (.50) | 100 (3) | 0 (0) |
| *Style* |  |  |  |
| - Cartoon | 4.75 (.50) | 66.7 (2) | 0 (0) |
| - Flat | 4.75 (.50) | 66.7 (2) | 25 (1) |
| - Hand drawn | 4.25 (.50) | 66.7 (2) | 50 (2) |

^1^Rated on scale of 1 (I hate it) to 5 (I love it). ^2^ missing data for one participant as they missed Workshop 1 so could not answer this question
