# Supplementary material for: Online Support and Intervention for Child Anxiety (OSI): Development and Usability Testing
Source: JMIR Form Res. 2022 Apr 13;6(4):e29846. doi: 10.2196/29846 (PMC9047721; doi:10.2196/29846)
Supplement: Multimedia Appendix 3 [file formative_v6i4e29846_app3.docx]

Appendix 3. Phase 1 children’s feedback on Online Support and Intervention for child anxiety game mock-ups

| Aspect of OSI game mock-up | Like^1^ (mean (SD)) | Ease of use^2^ (mean (SD)) | % (n) yes developer understood what wanted | % (n) want it changed |
| --- | --- | --- | --- | --- |
|  |  |  |  |  |
| Game character | 4.67 (.58) | N/A | 66.7 (2) | 0 (0) |
| Home screen | 4.33 (.58) | 3.67 (.58) | 66.7 (2) | 33.3 (1) |
| Character dress up game | 4.67 (.58) | 4.67 (.58) | 100 (3) | 33.3 (1) |
| Game selection screen | 4.00 (1.00) | 4.67 (.58) | 66.7 (2) | 66.7 (2) |
| Challenges screen | 5.00 (0) | 4.33 (.58) | 100 (3) | 0 (0) |
| Game option 1 | 4.67 (.58) | 4.00 (0) | 66.7 (2) | 33.3 (1) |
| Game option 2 | 4.33 (.58) | 4.67 (.58) | 100 (3) | 33.3 (1) |
| Game option 3 | 3.67 (.58) | 4.67 (.58) | 33.3 (1) | 66.7 (2) |
| Game option 4 | 4.33 (.58) | 4.33 (.58) | 100 (3) | 33.3 (1) |
| Game option 5 | 5.00 (0) | 4.33 (.58) | 66.7 (2) | 33.3 (1) |
| Game option 6 | 4.33 (.58) | 4.00 (0) | 66.7 (2) | 33.3 (1) |

^1^Rated on scale of 1 (I hate it) to 5 (I love it), ^2^Rated on scale of 1 (really hard) to 5 (easy)
