# Supplementary material for: Online Support and Intervention for Child Anxiety (OSI): Development and Usability Testing
Source: JMIR Form Res. 2022 Apr 13;6(4):e29846. doi: 10.2196/29846 (PMC9047721; doi:10.2196/29846)
Supplement: Multimedia Appendix 4 [file formative_v6i4e29846_app4.docx]

Multimedia Appendix 4. Phase 2 parents’ and clinicians’ feedback on working prototypes of Online Support and Intervention for child anxiety

| PCUQ item | Parents | | | Clinicians | | |
| --- | --- | --- | --- | --- | --- | --- |
|  | Iteration 1  mean (SD) | Iteration 2  mean (SD) | Iteration 3  mean (SD) | Iteration 1  mean (SD) | Iteration 2  mean (SD) | Iteration 3  mean (SD) |
|  |  |  |  |  |  |  |
| It is easy to use | 4.50 (.84) | 4.67 (.52) | 4.83 (.41) | 4.43 (.54) | 4.14 (.38) | 4.20 (.45) |
| It is easy to navigate | 4.50 (.84) | 4.50 (.84) | 5.00 (0) | 4.14 (.69) | 4.00 (0) | 3.80 (.84) |
| The words are clear and easy to understand | 4.50 (.84) | 5.00 (0) | 5.00 (0) | 4.00 (.58) | 4.29 (.49) | 4.40 (.55) |
| Each page (screen) has the right amount of information | 4.67 (.52) | 4.83 (.41) | 4.83 (.41) | 3.71 (.49) | 4.14 (.38) | 4.20 (.45) |
| Modules took an appropriate time to complete^1^ | 4.67 (.52) | 5.00 (0) | 4.83 (.41) | - | - | - |
| It is easy to use the menu bar | 4.50 (.84) | 4.50 (.84) | 4.83 (.41) | 4.14 (.39) | 4.29 (.49) | 4.00 (.71) |
| The buttons and menu options work as I expected them to | 4.20 (.84) | 4.50 (.55) | 4.83 (.41) | 3.86 (.69) | 4.43 (.79) | 4.00 (.71) |
| The site is visually pleasing to me | 4.00 (1.10) | 4.33 (.52) | 4.50 (.84) | 4.00 (.58) | 4.29 (.49) | 4.40 (.55) |
| It is always clear what to do next | 4.17 (.75) | 4.00 (.63) | 4.83 (.41) | 3.71 (.76) | 3.57 (.79) | 3.60 (.89) |
| The site is user-friendly | 4.33 (.82) | 4.83 (.41) | 4.67 (.82) | 4.14 (.38) | 4.00 (0) | 4.00 (0) |
| Each page (screen) loaded quickly | 4.50 (1.23) | 4.83 (.41) | 4.50 (1.23) | 4.86 (.38) | 4.33 (.52) | 4.40 (.89) |
| The video segments loaded quickly^1^ | 4.17 (1.33) | 5.00 (0) | 4.50 (.84) | - | - | - |
| I would return to this website | 4.83 (.41) | 5.00 (0) | 4.83 (.41) | 4.57 (.54) | 4.50 (.55) | 4.40 (.55) |
| The tone of the material is sensitive for parents seeking help for their child’s anxiety^1^ | 4.67 (.82) | 5.00 (0) | 4.83 (.41) | - | - | - |
| The material is relevant for parents seeking help for their child’s anxiety^1^ | 4.83 (.41) | 5.00 (0) | 5.00 (0) | - | - | - |

^1^Questionnaire item not relevant for clinicians to answer. Items were scored on a scale of 1 (strongly disagree) to 5 (strongly agree).
