# Supplementary material for: Online Support and Intervention for Child Anxiety (OSI): Development and Usability Testing
Source: JMIR Form Res. 2022 Apr 13;6(4):e29846. doi: 10.2196/29846 (PMC9047721; doi:10.2196/29846)
Supplement: Multimedia Appendix 5 [file formative_v6i4e29846_app5.docx]

Multimedia Appendix 5. Phase 2 children’s feedback on working prototypes of the game

| PCUQ item | Iteration 1  mean (SD) | Iteration 2  mean (SD) | Iteration 3  mean (SD) |
| --- | --- | --- | --- |
|  |  |  |  |
| It is easy to use | 3.75 (.50) | 4.25 (.96) | 4.50 (.58) |
| The words are easy to understand | 5.00 (0) | 5.00 (0) | 4.75 (.50) |
| Each page (screen) has the right amount of information | 4.25 (.96) | 4.25 (.50) | 3.75 (.96) |
| It is easy to use the home screen | 4.50 (1.00) | 4.00 (.82) | 3.75 (1.26) |
| The buttons work as I expected them to | 4.75 (.50) | 4.25 (.96) | 3.75 (1.26) |
| The game looks good | 4.50 (.58) | 4.50 (.58) | 4.50 (1.00) |
| It is always clear what to do next | 4.50 (.58) | 3.75 (1.26) | 4.00 (.82) |
| The game is user-friendly | 4.50 (1.00) | 4.75 (.50) | 5.00 (0) |
| Each page (screen) loaded quickly | 4.75 (.50) | 3.50 (1.73) | 3.25 (.96) |
| I would use this game again | 4.25 (1.50) | 4.00 (2.00) | 4.25 (.96) |
| This game would be good for children who are getting help for their anxiety | 4.00 (2.00) | 4.25 (1.50) | 4.00 (1.41) |

Items were rated on scale of 1 (I hate it) to 5 (I love it)
