# Supplementary material for: Online Support and Intervention for Child Anxiety (OSI): Development and Usability Testing
Source: JMIR Form Res. 2022 Apr 13;6(4):e29846. doi: 10.2196/29846 (PMC9047721; doi:10.2196/29846)
Supplement: Multimedia Appendix 7 [file formative_v6i4e29846_app7.pptx]

## Slide 1
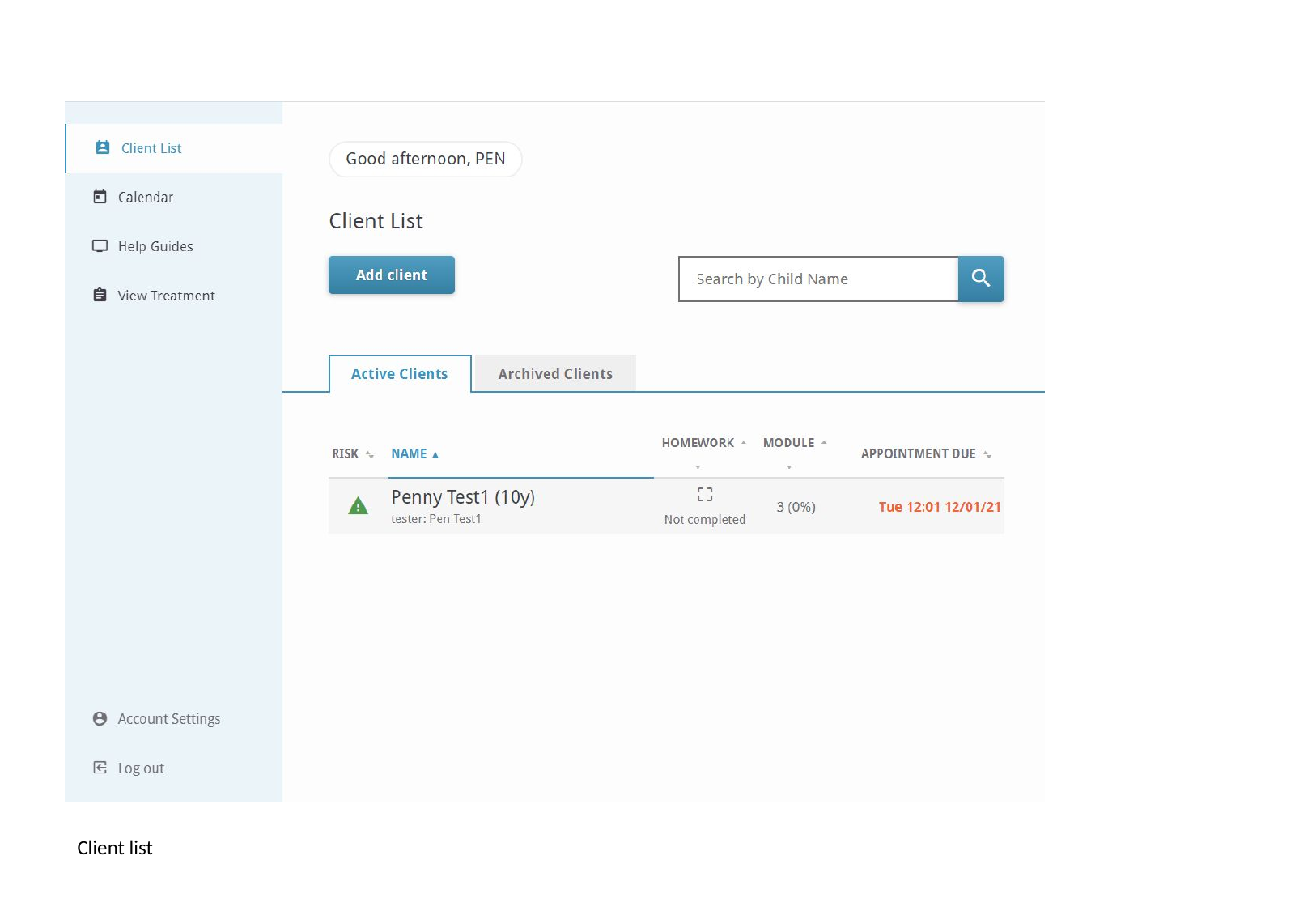

Client list

## Slide 2
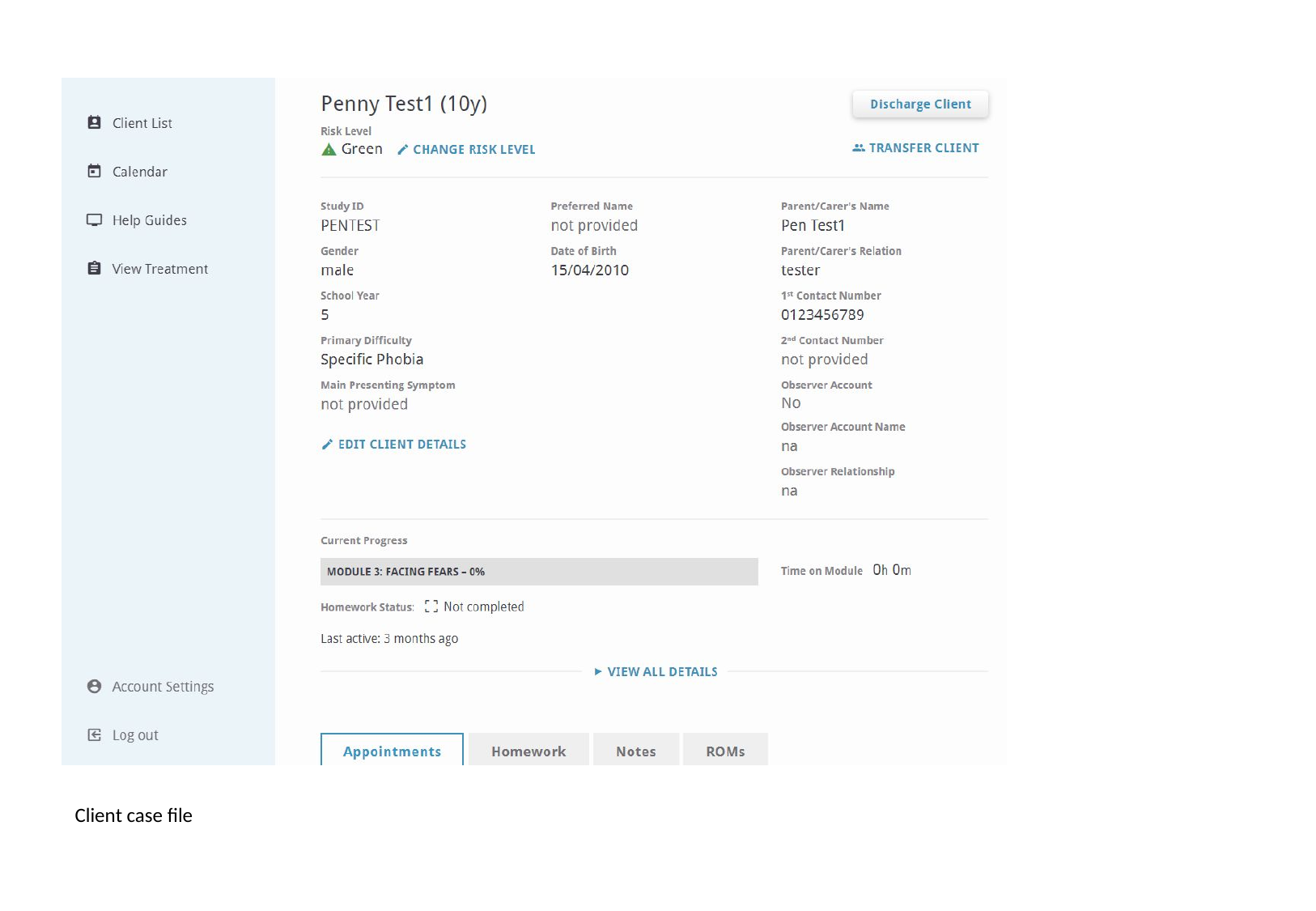

Client case file

## Slide 3
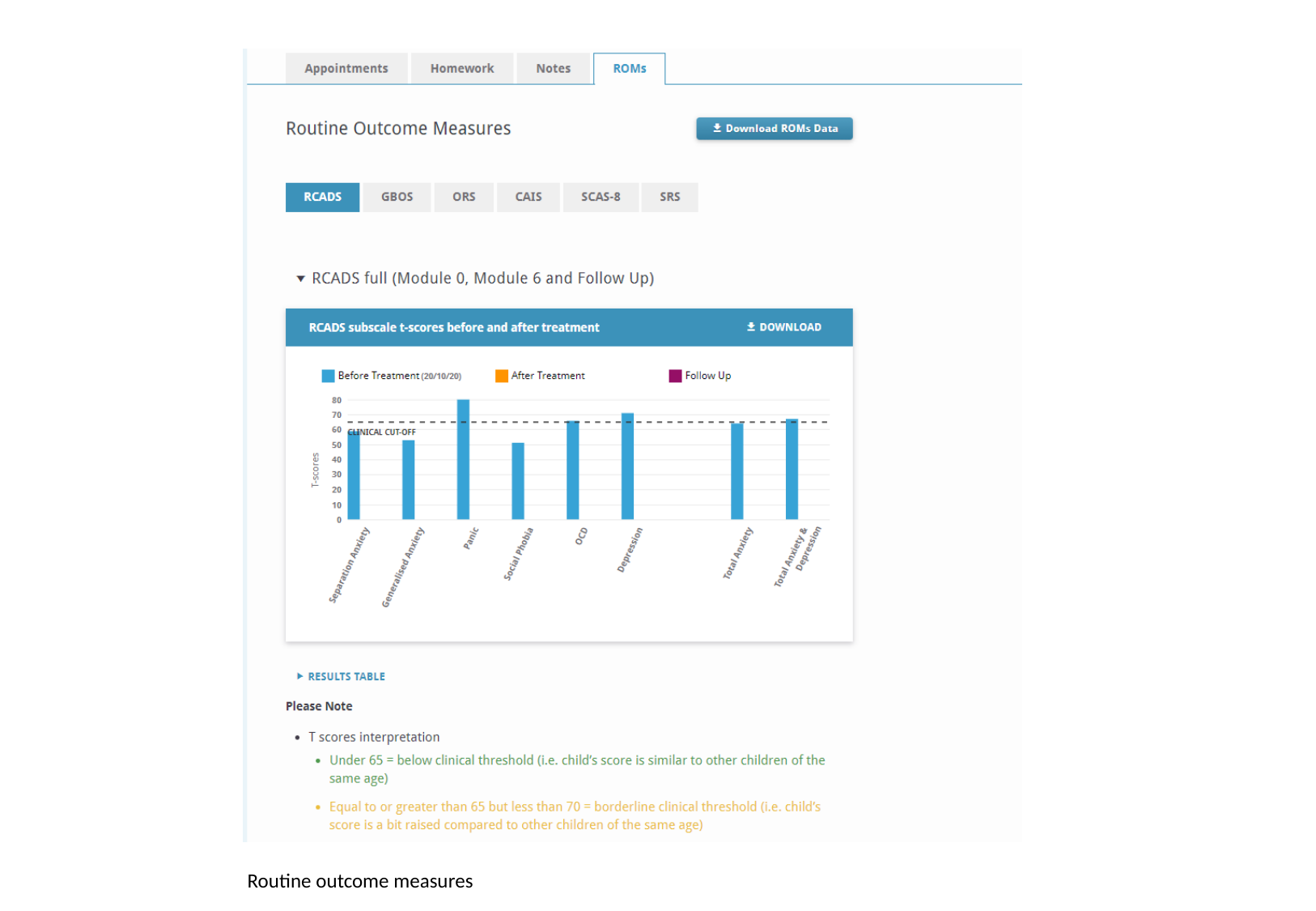

Routine outcome measures

## Slide 4
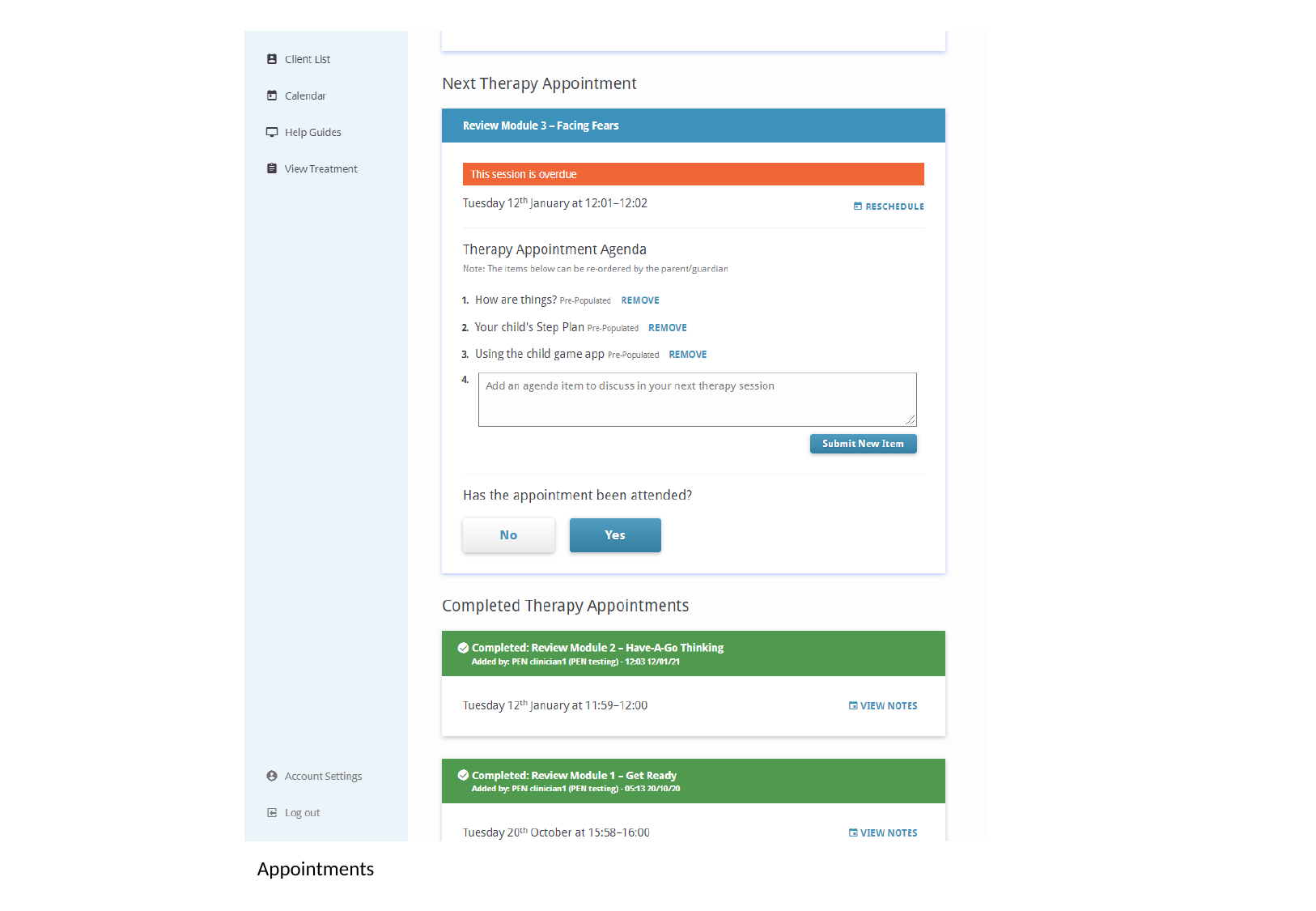

Appointments

## Slide 5
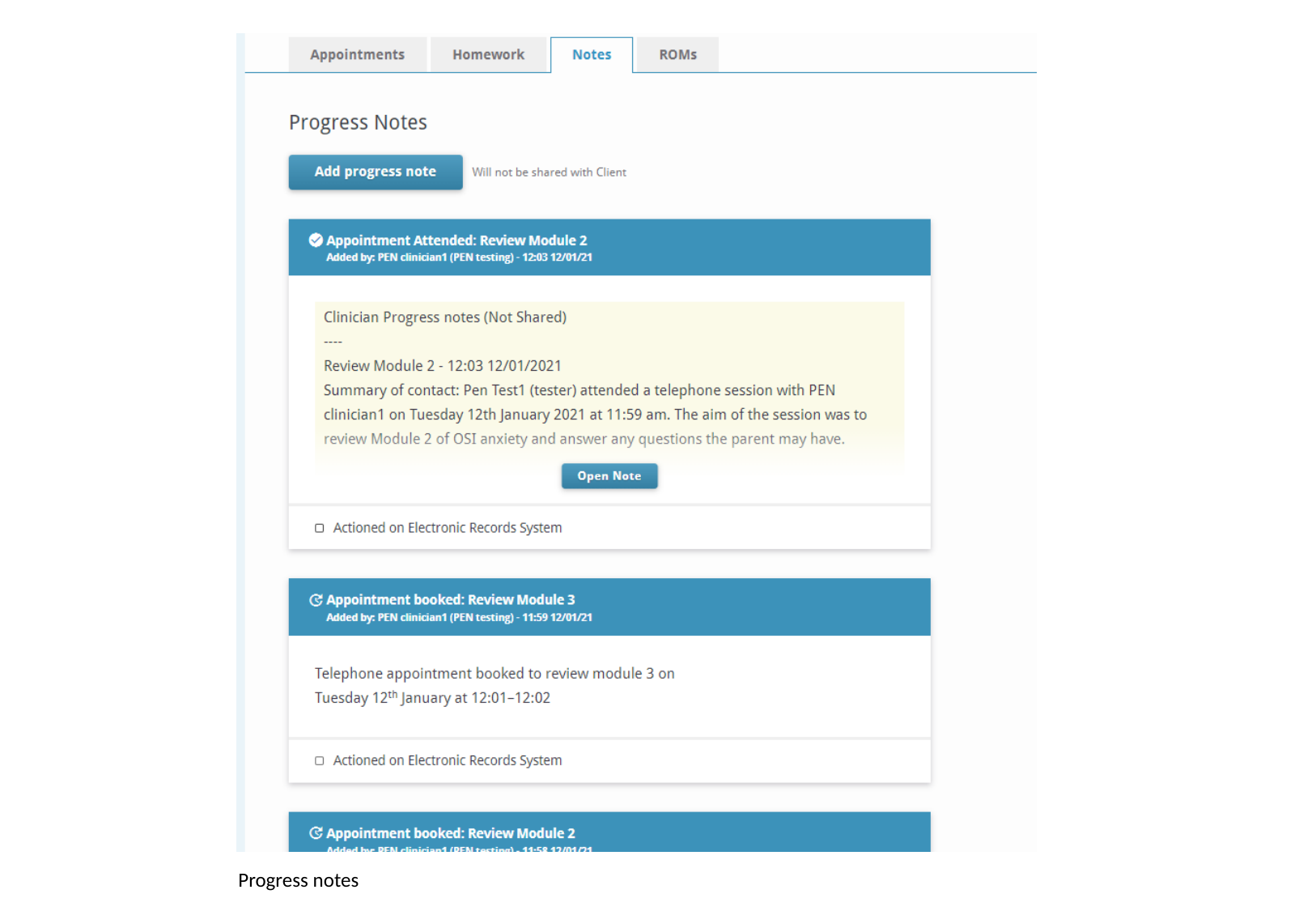

Progress notes

## Slide 6
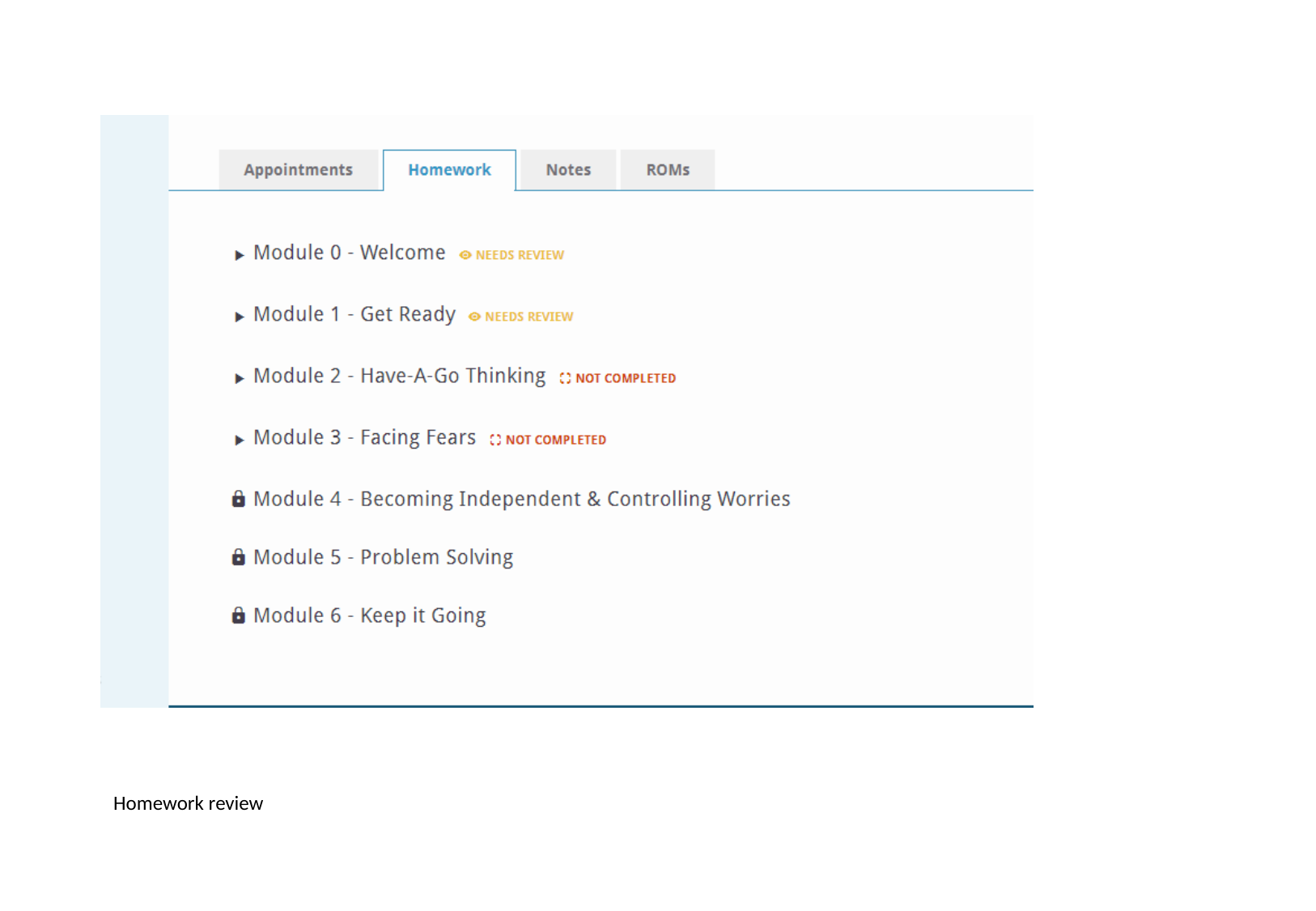

Homework review
