# Supplementary figures and images for: Online Support and Intervention for Child Anxiety (OSI): Development and Usability Testing
Source: JMIR Form Res. 2022 Apr 13;6(4):e29846. doi: 10.2196/29846 (PMC9047721; doi:10.2196/29846)

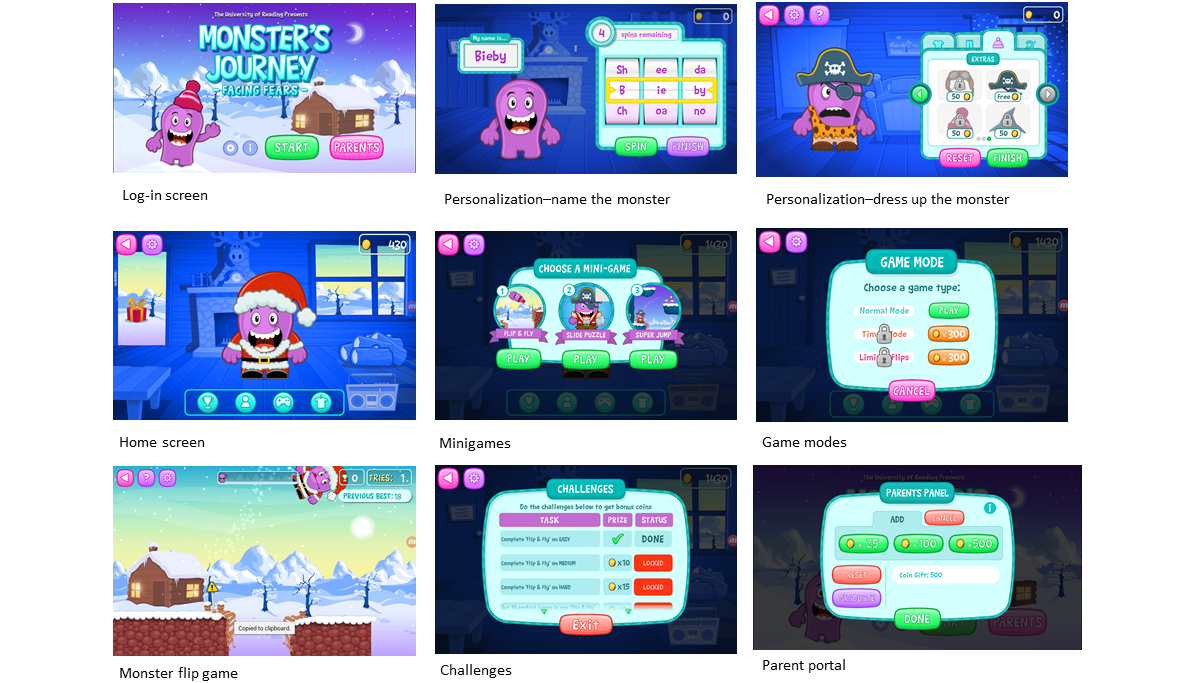

Supplement: Multimedia Appendix 8 [file formative_v6i4e29846_app8.png]
