# Supplementary material for: Online Support and Intervention for Child Anxiety (OSI): Development and Usability Testing
Source: JMIR Form Res. 2022 Apr 13;6(4):e29846. doi: 10.2196/29846 (PMC9047721; doi:10.2196/29846)
Supplement: Multimedia Appendix 9 [file formative_v6i4e29846_app9.docx]

Multimedia Appendix 9. Online Support and Intervention for child anxiety treatment content

| **Module** | **Topics covered** |
| --- | --- |
|  |  |
| Module 0: Welcome | Overview of treatment and how to use OSI |
| Module 1: Get Ready | Psychoeducation and goal setting |
| Module 2: Have-A-Go Thinking | Cognitive restructuring |
| Module 3: Facing Fears | Exposure |
| Module 4: Becoming Independent & Controlling Worries | Excessive worry management and increasing independence |
| Module 5: Problem Solving | Problem solving |
| Module 6: Keep it Going | Relapse prevention |
| Follow Up | ROMS only - no new content presented |
